# Elevated α-hydroxybutyrate dehydrogenase is associated with in-hospital mortality in non-ischemic dilated cardiomyopathy

**DOI:** 10.3389/fcvm.2022.995899

**Published:** 2022-09-20

**Authors:** Xinyi Li, Wenfei He, Xiaonan Zhang, Fen Shu, Yaoxin Liu, Ning Tan, Lei Jiang

**Affiliations:** ^1^Department of Cardiology, Guangdong Provincial Geriatrics Institute, Guangdong Provincial People's Hospital, Guangdong Academy of Medical Sciences, School of Medicine, South China University of Technology, Guangzhou, China; ^2^Department of Cardiology, Guangdong Provincial People's Hospital's Nanhai Hospital, The Second People's Hospital of Nanhai District, Foshan, China; ^3^Department of Cardiology, Guangdong Cardiovascular Institute, Guangdong Provincial People's Hospital, Guangdong Academy of Medical Sciences, Guangzhou, China

**Keywords:** NIDCM, elderly, α-HBDH, in-hospital death, prognosis

## Abstract

**Background:**

Previous Study Found That Implantation of a Cardioverter-Defibrillator Likely Caused a Worse Prognosis in Older Patients With non-Ischemic Systolic Heart Failure. This Suggests That More Precise Risk Stratification Is Needed in Elderly Patients. We Conducted a Retrospective Study to Evaluate the Association of α-Hydroxybutyrate Dehydrogenase (α-HBDH) With Mortality During Hospitalization in Elderly Patients With non-Ischemic Dilated Cardiomyopathy (NIDCM).

**Methods:**

1,019 Elderly Patients (age ≥60 Years) Diagnosed With NIDCM Were Retrospectively Enrolled From January 2010 to December 2019. Univariate and Multivariate Analyses Were Showed to Explore the Relationship Between α-HBDH and in- Hospital Death.

**Results:**

Patients in elevated α-HBDH group (>182 U/L) had a longer hospital stays and higher in-hospital mortality. Univariate logistics regression analysis showed that elevated α-HBDH was significantly related to mortality (OR: 7.004, 95% CI: 3.583–13.693, *p* < 0.001). Receiver operator characteristic (ROC) curve analysis reflected that α-HBDH levels had excellent predictive power for in-hospital death (AUC = 0.810, 95% CI: 0.745–0.876, *p* < 0.001). After adjustment of age, serum creatine, albumin and LVEF, multivariate regression analysis validated the association of elevated α-HBDH with increased risk of in-hospital death (*p* < 0.05).

**Conclusions:**

Elevated α-HBDH level is significantly related to in-hospital mortality in older patients with NIDCM.

## Introduction

Non-Ischemic Dilated Cardiomyopathy (NIDCM) Is Defined as Left Ventricle (LV) Enlargement and Global Systolic Function Impairment (LVEF <45%) in the Absence of Coronary Artery Disease or Increased Loading Condition (1). Currently, There Are no Effective Treatments can Prevent the Progression of NIDCM to HF (2). These Patients Often Occur Refractory Heart Failure (HF) and Sudden Cardiac Death (SCD) and Need Benefit From Cardiac Transplantation (3–5). This Condition Brings Great Pain to Patients and Places a Heavy Financial Burden on Global Health Care Systems (6, 7). The DANISH Study Found That Older Patients With non-Ischemic Systolic HF may not Benefit From Implantation of a Cardioverter-Defibrillator (8). This Suggests That More Precise Risk Stratification Is Needed in Elderly Patients.

α-hydroxybutyrate dehydrogenase (α-HBDH) is an auxiliary marker of myocardial injury, which was reported to have an increased specificity for detecting myocardial injury, starting to increase 8–12 h after damage, reaching peak serum concentrations after 48–72 h and returning to baseline after 7–14 days (9). Myocardial injury was associated with progression and poor outcomes of DCM (10). Therefore, we aimed to explore the connection between α-HBDH and in-hospital mortality in elderly patients with NIDCM.

## Materials and methods

### Study population

We retrospectively enrolled 1,019 patients (age ≥60 years) admitted for NIDCM in Guangdong Provincial People's Hospital (Guangzhou, China) from January 2010 to December 2019. All patients met the diagnostic criteria of NIDCM according to the scientific statement established by the American Heart Association (11). This study received approval of the Ethics Committee of Guangdong Provincial People's Hospital with a waiver of written informed consent due to the retrospective study design. Oral informed consent from patients or their relatives by telephone was recorded by trained nurses during follow-up.

### Data source

Clinical information and laboratory results were collected from the electronic medical records by one researcher and randomly confirmed by another researcher. Basal α-HBDH samples were collected on the following morning after admission and measured by colorimetric method. The LV ejection fraction (LVEF) was measured by Simpson's biplane method. Linear internal measurements of the left ventricle and its walls were completed in the parasternal view.

### Definition and endpoints

Elevated α-HBDH on admission was defined as a α-HBDH level of more than 182 U/L according to the standards established by our laboratory. There were no patients had reduced α-HBDH (<72 U/L). The primary endpoint was in-hospital death, followed by malignant arrhythmia and acute HF during hospitalization. Malignant arrhythmia included ventricular tachycardia, ventricular fibrillation, and sudden cardiac arrest.

### Statistical analysis

Continuous data are expressed as the mean ± SD, compared by One-way ANOVA tests. Categorical variables are presented as numbers, *n* (proportions, %) and compared through Pearson's chi-square tests. Missing values were excluded from the analysis. Univariate and multivariate analyses were performed to evaluate the association of elevated α-HBDH level with in-hospital mortality. Receiver operator characteristic (ROC) curves were drawn to assess the predictive power of α-HBDH. All statistical analyses were performed using SPSS software version 26.0 (IBM Corp., Armonk, New York, USA). All *P*-values were two sided with a significance level of 0.05.

## Results

### Baseline characteristics

There are 1,019 patients (667 males, 352 females) met the inclusion criteria and were divided into two groups according to the α-HBDH levels on admission: normal α-HBDH (≤ 182 U/L) and elevated α-HBDH (>182 U/L). In the two groups, the range of age was 60–91 and 60–89 years, respectively, and there were 151 patients older than 75 years old. The mean age and sex composition were similar.

First, the medical history of smoking, diabetes, and hypertension were similar in the two groups. Second, patients with elevated α-HBDH had a higher lactate dehydrogenase (LDH) and lower albumin level and LVEF on admission. Third, there was no statistic difference in the prevalence of malignant arrhythmia and acute HF during hospitalization. Patients in elevated α-HBDH group performed a longer mean hospital stays (11 vs. 8 days) and higher mortality during hospitalization (10.9 vs. 1.7%) (Table 1).

**Table 1:** Baseline demographics and clinical characteristics.

**Variables**	**Patients with NIDCM (*****N*** = **1,019)**		***P*-value**
	**Normal α-HBDH**	**Elevated α-HBDH**	
	**(*n* = 754)**	**(*n* = 265)**	
**Demographic**
Age, y	68.1 ± 6.1	68.8 ± 6.6	0.124
Male, *n* (%)	503 (66.7)	164 (61.9)	0.155
**Medical history**
Smoking history, *n* (%)	211 (28)	61 (23)	0.116
Hypertension, *n* (%)	258 (34.2)	82 (30.9)	0.331
Diabetes, *n* (%)	175 (23.2)	73 (27.5)	0.157
**Parameters on admission**
WBC count, 109/L	7.6 ± 3.0	7.3 ± 2.3	0.087
Neutrophil count, 109/L	5.1 ± 2.7	4.8 ± 2.1	0.060
Lymphocyte count, 109/L	1.7 ± 1.3	1.6 ± 0.7	0.939
Hemoglobin, g/L	131.8 ± 18.7	130.4 ± 18.9	0.318
Platelet count, 109/L	198.0 ± 67.3	197.2 ± 69.8	0.878
Albumin, g/L	36.0 ± 4.5	33.5 ± 4.8	<0.001
CREA, umol/L	103.8 ± 68.3	136.9 ± 86.9	<0.001
ALT, U/L	27.3 ± 27.6	158.6 ± 460.2	<0.001
AST, U/L	29.1 ± 28.1	214.8 ± 1,196.5	<0.001
LDH, U/L	189 ± 38	430 ± 621	<0.001
α-HBDH, U/L	137 ± 26	255 ± 88	<0.001
Total bilirubin, umol/L	18.8 ± 10.4	34.3 ± 40.3	<0.001
PAP, mmHg	40.4 ± 14.4	45.7 ± 14.3	<0.001
LVEF, %	33.8 ± 11.2	31.2 ± 10.6	0.003
Hospital stays, days	8 ± 6	11 ± 9	<0.001
**Clinical symptoms and outcomes**
Malignant arrhythmia, *n* (%)	51 (6.8)	25 (9.4)	0.155
Acute heart failure, *n* (%)	10 (1.3)	4 (1.5)	0.826
In-hospital mortality, *n* (%)	13 (1.7)	29 (10.9)	<0.001

NIDCM, non-ischemic dilated cardiomyopathy; WBC, white blood cell; CREA, creatinine; ALT, alanine aminotransferase; AST, aspartate aminotransferase; PAP, pulmonary artery pressure; LVEF, left ventricle ejection fraction.

### Regression analyses and ROC curve

Univariate logistics regression analysis showed that elevated α-HBDH was not related to malignant arrhythmia and acute HF in hospital (both *p* > 0.05), but it was significantly associated with mortality (OR: 7.004, 95% CI: 3.583–13.693, *p* < 0.001). Multivariate logistics regression for in-hospital death and α-HBDH displayed statistic difference in the two groups (OR: 4.217, 95% CI: 1.958–9.082, *p* < 0.001) (Table 2). Meanwhile, multivariate Cox regression analysis confirmed the relationship between elevated α-HBDH and increased risk of in-hospital death (HR: 2.489, 95% CI: 1.217–5.089, *p* = 0.012) (Table 3). The adjustment variables enrolled in the multivariate regression model were age, serum creatine, albumin and LVEF, whose *p*-value was <0.05 in univariate regression analysis.

**Table 2:** The risk of elevated α-hydroxybutyrate dehydrogenase for in-hospital death during hospitalization.

	**Group**	**Logistic regression analysis**		
		**OR**	**95% CI**	***P*-value**
Univariate analysis	Normal α-HBDH	Ref	/	/
	Elevated α-HBDH	7.004	3.583–13.693	<0.001
Multivariate analysis^a^	Normal α-HBDH	Ref	/	/
	Elevated α-HBDH	4.217	1.958–9.082	<0.001

OR, odds ratio; CI, confidence interval. ^a^Adjust for age, serum creatine, albumin, and left ventricle ejection fraction.

**Table 3:** The risk of elevated α-hydroxybutyrate dehydrogenase for in-hospital death during hospitalization.

	**Group**	**Cox regression analysis**		
		**HR**	**95% CI**	***P*-value**
Univariate analysis	Normal α-HBDH	Ref	/	/
	Elevated α-HBDH	4.052	2.069–7.934	<0.001
Multivariate analysis^a^	Normal α-HBDH	Ref	/	/
	Elevated α-HBDH	2.489	1.217–5.089	0.012

HR, hazard ratio; CI, confidence interval. ^a^Adjust for age, serum creatine, albumin, and left ventricle ejection fraction.

**Figure 1:**
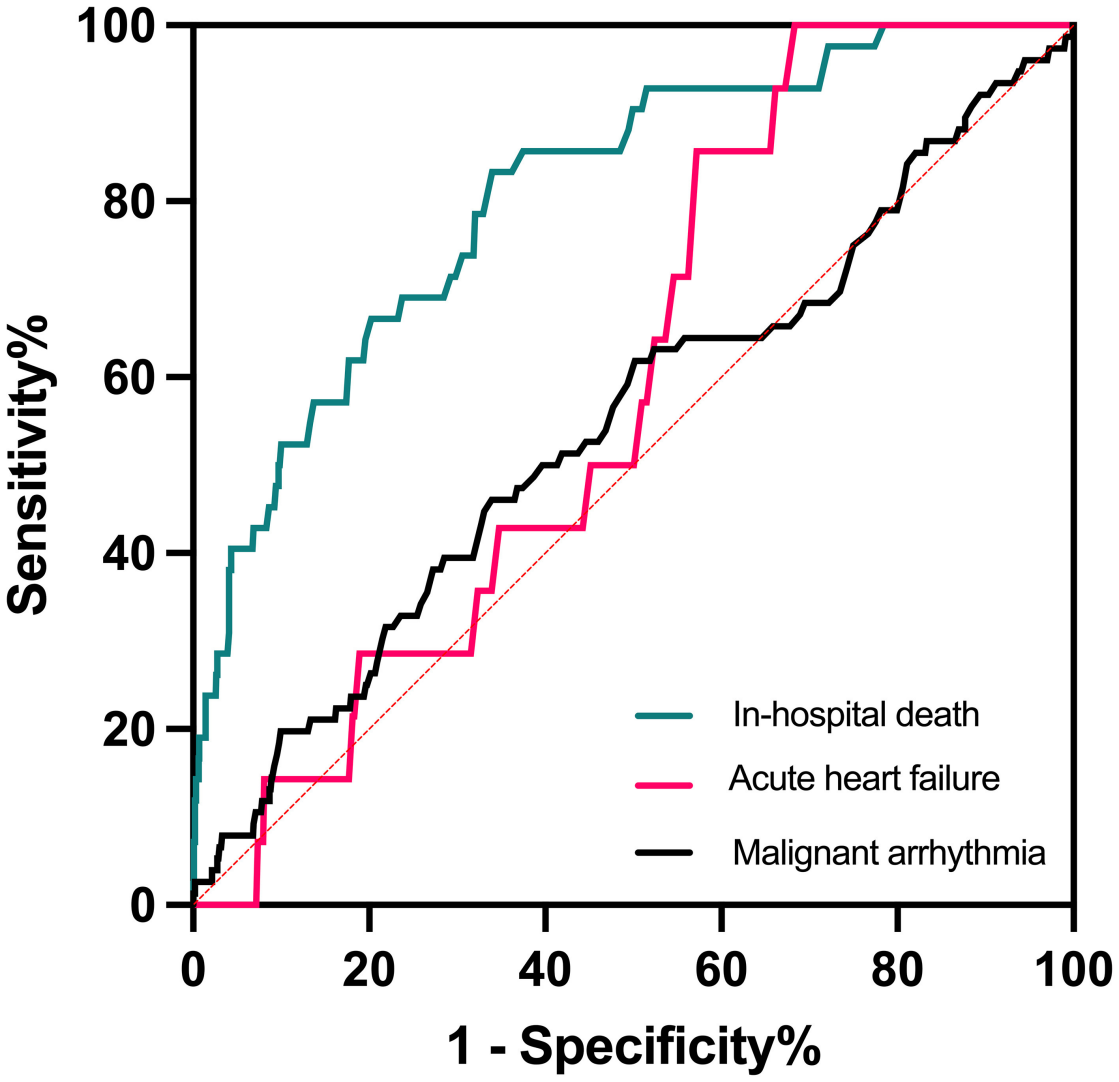
ROC curves of α-HBDH in predicting in-hospital endpoints.

ROC curve analysis showed that α-HBDH levels had great sensitivity and specificity in predicting mortality (AUC = 0.810, 95% CI: 0.745–0.876, *p* < 0.001) but not malignant arrhythmia and acute HF (both *p* > 0.05) during hospitalization. The area under the curve (AUC) is a measure of the overall predictive validity of the risk and prognosis in the disease where AUC >0.80 signals good validity. The optimal cutoff value was 168 U/L, with a sensitivity of 83.3% and specificity of 66.0% (Figure 1).

## Discussion

At present, this is the largest and first study to investigate the connection between α-HBDH levels and in-hospital mortality in patients with NIDCM. The results showed that elevated α-HBDH was an independent risk factor for in-hospital death in elderly patients with NIDCM, which may serve as a potential therapeutic target for reducing mortality during hospitalization.

Previous study has reported that α-HBDH have an increased specificity for detecting myocardial injury (9). NIDCM is characterized by LV enlargement and global systolic function impairment, and often accompanied by myocardial injury (1). Older age is always accompanied by deceleration of cell metabolism, and more extensive damage to organ. α-HBDH is significantly associated with age as a marker of cell death (12). In the present study, we selected a cohort of elderly people (age ≥60 years) and divided them into two groups. There was no statistic difference in age and sex composition between the two groups. However, patients with elevated α-HBDH had a higher rate of in-hospital mortality than those with normal α-HBDH.

The present study showed that patients in elevated group had elevated LDH, and reduced albumin level and LVEF on admission. Changes in α-HBDH levels and these markers may affect mutually, such as reduced albumin level indicates malnutrition (13) and lower LVEF is associated with damage of systolic function of myocardium (14). All these changes are related to poor outcome in patients. It also indicates that the relationship between elevated α-HBDH and poor prognosis is associated with the abnormal situations of several systems. However, future studies are required to elucidate the specific mechanism.

The molecular basis of the irregular cardiac architecture in NIDCM involves changes in the structure and composition of cardiomyocytes, which lead to remodeling of the myocardium (15). Myocardial remodeling may cause the phenotype of a patchwork LV, where myocardial cells are interspersed with necrotic and fibrotic patches and intermittent calcifications (4, 16). These mechanisms increase dilatation of the ventricle and are responsible for the reduction of LVEF, and, consecutively, clinical symptoms (17). Patients with NIDCM are often admitted to the hospital because of HF. Current guidelines suggest that treatment for these patients is mostly supportive general HF treatment (11). Patients often receive the therapy with β-blockers and angiotensin-converting enzyme inhibitors or angiotensin II-receptor blockers (ACEIs/ARBs), which has been proved to the survival of patients in clinical trials (18, 19). However, such patients remain at substantial risk for sudden death from cardiac causes. It is essential to early identify high-risk patients during hospitalization.

α-HBDH is a marker of apoptosis, which can reflect myocardial and renal damage (9). Patients in elevated α-HBDH group had higher serum creatine and lower LVEF. It was reported that α-HBDH was related to the occurrence of atherothrombotic events (20). In the present study, we had excluded patients with ischemic heart disease. The result showed that α-HBDH remained significantly associated with mortality during hospitalization in non-ischemic patients, but not malignant arrhythmia and acute HF. In addition, patients with elevated α-HBDH had longer hospital stays and higher in-hospital mortality (10.9 vs. 1.7%). The underlying mechanism is not clear and future trials are warranted.

The α-HBDH/LDH ratio between 0.63 and 0.81 is normal, while more than 0.9 suggests myocardial lesion, <0.6 suggests liver damage (12). The ratio α-HBDH/LDH in elevated α-HBDH group is 0.59 (255/430, Table 1), but normal in another group (137/189). In addition, the AST and ALT levels were abnormally higher and AST/ALT ratio was more than 1 (214/158, Table 1) in elevated group. This condition indicates that those patients are likely to have severe liver damage or even liver cancer. This suggests that the relationship between α-HBDH and mortality during hospitalization may be related to multiple organ damage, including heart, liver, kidney, and others.

Patients with NIDCM are usually admitted because of severe HF symptoms. At present, ACEIs/ARBs and β-blockers are the optimal medical therapy for HF in DCM (4). However, in acute and severe patients, pharmacological therapy may not be sufficient to maintain adequate cardiac function, which will increase the risk of in-hospital death. Those patients may need to benefit from cardiac resynchronization therapy (CRT) or even surgery. In a meta-analysis, CRT was proved to reduce mortality in non-ischemic cardiomyopathy (21). Surgical treatment mainly involves heart transplantation and implantation of long-term mechanical circulatory support (19). The results of this study suggest that elevated α-HBDH on admission was related to higher risk of death, which may help clinicians select the optimal treatment for patients and reduce their in-hospital mortality.

Some limitations should be declared in this research project. First, potential confounding factors may affect the results due to the retrospective study design. Second, α-HBDH levels are measured on admission and the influence of continuous monitoring cannot be ignored. Third, we did not correct for the effect of diuretics and other drugs on outcome.

In a word, we discovered that α-HBDH levels may be an independent risk factor for in-hospital death in elderly patients with NIDCM. Early monitoring of α-HBDH may help identify high risk patients and conduct beneficial intervention.

## Data availability statement

The data analyzed in this study is subject to the following licenses/restrictions: The datasets generated and/or analyzed during the current study are not publicly available due to privacy or ethical restrictions but are available from the corresponding author on reasonable request. Requests to access these datasets should be directed to jianglei@smu.edu.cn.

## Ethics statement

The studies involving human participants were reviewed and approved by Ethics Committee of Guangdong Provincial People's Hospital. Written informed consent for participation was not required for this study in accordance with the national legislation and the institutional requirements.

## Author contributions

LJ contributed to the design and conception of the study. XL, WH, XZ, FS, and YL were responsible for the acquisition, analysis, and interpretation of data. XL and WH drafted the manuscript. LJ and NT critically reviewed the manuscript. All authors gave final approval and agreed to be accountable for all aspects of work ensuring integrity and accuracy.

## Funding

This study was supported by National Natural Science Foundation (Grant Nos. 82170339 and 82270241), NSFC Incubation Project of Guangdong Provincial People's Hospital (Grant No. KY0120220021), Science and Technology Planning Project of Guangzhou (Grant No. 202102080055), and Medical Science and Technology Research Fund Project of Guangdong (Grant No. C2020005).

## Conflict of interest

The authors declare that the research was conducted in the absence of any commercial or financial relationships that could be construed as a potential conflict of interest.

## Publisher's note

All claims expressed in this article are solely those of the authors and do not necessarily represent those of their affiliated organizations, or those of the publisher, the editors and the reviewers. Any product that may be evaluated in this article, or claim that may be made by its manufacturer, is not guaranteed or endorsed by the publisher.
